# *N*-Formyl Peptide Receptors Induce Radical Oxygen Production in Fibroblasts Derived From Systemic Sclerosis by Interacting With a Cleaved Form of Urokinase Receptor

**DOI:** 10.3389/fimmu.2018.00574

**Published:** 2018-04-04

**Authors:** Filomena Napolitano, Francesca Wanda Rossi, Ada Pesapane, Silvia Varricchio, Gennaro Ilardi, Massimo Mascolo, Stefania Staibano, Antonio Lavecchia, Pia Ragno, Carmine Selleri, Gianni Marone, Marco Matucci-Cerinic, Amato de Paulis, Nunzia Montuori

**Affiliations:** ^1^Department of Translational Medical Sciences, University of Naples Federico II, Naples, Italy; ^2^Center for Basic and Clinical Immunology Research (CISI), WAO Center of Excellence, University of Naples Federico II, Naples, Italy; ^3^Department of Advanced Functional Sciences, Pathology Section, University of Naples Federico II, Naples, Italy; ^4^Department of Pharmacy, Drug Discovery Laboratory, University of Naples Federico II, Naples, Italy; ^5^Department of Chemistry and Biology, University of Salerno, Salerno, Italy; ^6^Department of Medicine and Surgery, University of Salerno, Salerno, Italy; ^7^Institute of Experimental Endocrinology and Oncology (IEOS), Consiglio Nazionale delle Ricerche (CNR), Naples, Italy; ^8^Department of Experimental and Clinical Medicine, University of Florence, Florence, Italy; ^9^Department of Geriatric Medicine, Division of Rheumatology AOUC, University of Florence, Florence, Italy

**Keywords:** inflammation, fibrosis, systemic sclerosis, FPRs, uPAR, integrin, ROS, fibroblasts

## Abstract

Systemic sclerosis (SSc) is a chronic autoimmune disease characterized by fibrosis, alteration in the microvasculature and immunologic abnormalities. It has been hypothesized that an abnormal redox state could regulate the persistent fibrotic phenotype in SSc patients. *N*-Formyl peptide receptors (FPRs) are chemotactic receptors overexpressed in fibroblasts derived from SSc patients. In this study, we demonstrated that stimulation of FPRs promotes the generation of reactive oxygen species (ROS) in skin fibroblasts. In fibroblast cells, ROS production was due to FPRs interaction with the urokinase receptor (uPAR) and to β_1_ integrin engagement. FPRs cross-talk with uPAR and integrins led to Rac1 and ERKs activation. FPRs stimulation increased gp91phox and p67phox expression as well as the direct interaction between GTP-Rac1 and p67phox, thus promoting assembly and activation of the NADPH oxidase complex. FPRs functions occur through interaction with a specific domain of uPAR (residues _88_SRSRY_92_) that can be exposed on the cell membrane by protease-mediated receptor cleavage. Immunohistochemistry analysis with a specific anti-SRSRY antibody showed increased expression of uPAR in a cleaved form, which exposes the SRSRY sequence at its N-terminus (DIIDIII-uPAR88–92) in skin biopsies from SSc patients. As expected by the increased expression of both FPRs and DII-DIII-uPAR_88-92_, fibroblasts derived from SSc patients showed a significantly increase in ROS generation both at a basal level than after FPRs stimulation, as compared to fibroblasts from normal subjects. C37, a small molecule blocking the interaction between FPRs and uPAR, and selumetinib, a clinically approved MAPKK/ERK inhibitor, significantly inhibited FPRs-mediated ROS production in fibroblasts derived from SSc patients. Thus, FPRs, through the interaction with the uPA/uPAR system, can induce ROS generation in fibroblasts by activating the NADPH oxidase, playing a role in the alteration of the redox state observed in SSc.

## Introduction

Systemic sclerosis (SSc) is an autoimmune disorder characterized by thickening of the skin and a severe and often progressive fibrosis of multiple internal organs. Fibrosis is a process characterized by a deregulated repair with an excessive deposition of collagen and other extracellular matrix components, which follows the features of the embryonic development and of the physiological wound healing (1, 2).

The release of reactive oxygen species (ROS) (3) as well as the secretion of chemokines and growth factors, i.e., platelet-derived growth factor (PDGF), transforming growth factor beta (TGF-β), connective tissue growth factor (CTGF), interleukin-6 (IL-6), and interleukin-13 (IL-13), all significantly higher in SSc patients than in controls (1–3), can promote fibroblast activation, fibroblast to myofibroblast transition, and collagen deposition in fibrosis (4).

High levels of ROS and oxidative stress have been directly or indirectly implicated in SSc (1). Indeed, free radicals through a direct profibrogenic effect on fibroblasts contribute to the production of key factors implicated in fibrosis, such as TGF-β (1–4).

In SSc, high levels of ROS are observed in fibroblasts, due to the stimulation of the membrane NADPH oxidase system (5–7). Early in the disease, inflammation generates a mild oxidative stress. Low levels of ROS stabilize and increase the Ras protein level that, in turn, determines increased sensitivity of fibroblast cells to growth factors. ROS also inhibits tyrosine phosphatases and maintains MEK, and ERK1/2 in the active state. Subunits p67 and p47 of the NADPH oxidase undergo phosphorylation by ERK1/2 and further stimulate the production of ROS (1, 8). In addition, a ROS-mediated loop increases the expression of NADPH oxidase 2 and 4 in skin fibroblasts from SSc patients (9).

The circuit linking Ras with ERK1/2 and ROS amplifies and maintains the cytokines and growth factors and their cognate receptors in an autocrine amplification loop (1).

*N*-formyl peptide receptors (FPRs) are a family of pattern recognition receptors, regulating innate responses (10). FPRs, by interacting with several structurally diverse pro- and anti-inflammatory ligands, possess important regulatory effects in multiple pathophysiological conditions, including inflammation and cancer (10, 11).

We have previously demonstrated that skin fibroblasts from SSc patients overexpress all the three FPRs (FPR1, FPR2, and FPR3) (12). Leoni et al. recently identified a novel intestinal epithelial FPR signaling pathway that is activated by an endogenous FPR1 ligand, annexin A1, and its cleavage product Ac2-26, which determines generation of ROS through NOX1, an epithelial NADPH oxidase (13). The redox signaling pathway resulting from the epithelial FPR1/NOX-1-activation promotes mucosal wound repair.

Since inappropriate NADPH oxidase activation seems to play a fundamental role in determining fibrosis in SSc (1, 4, 8, 9), and FPRs are able to activate NADPH oxidase, both in leukocytes (14) and epithelial cells (13), we sought to investigate whether FPRs could be involved in ROS generation in fibroblasts derived from normal and SSc subjects, through the interaction with the urokinase (uPA)/urokinase receptor (uPAR) system.

Indeed, several functions of FPRs occur through the interaction with uPAR. uPAR is characterized by three homologous domains (DI, DII, DIII) anchored to the cell membrane by a glycosyl-phosphatidylinositol (GPI) tail. uPAR is able to interact with FPRs, tyrosine kinase receptors and integrins, regulating main signal transduction pathways engaged in wound repair, angiogenesis, and tumor progression (15). In the flexible linker connecting uPAR domains DI and DII, a specific region of uPAR, corresponding to amino acids 88–92 (SRSRY), interacts with FPRs, mediating uPA or fMLF-dependent cell migration. uPA or its aminoterminal fragment (ATF), upon binding to the receptor, can promote uPAR interaction with FPRs, through the exposure of the uPAR_88–92_ region. Moreover, the removal of DI, uPA-mediated, results in the expression on the cell surface of a truncated uPAR form that can contain the chemotactic peptide that is able to interact with FPRs and to regulate their signal (DII-DIII-uPAR_88–92_) (16).

Besides FPRs, uPAR can also interact with other cell surface receptors, such as integrins and receptor tyrosine kinases (16–22). uPAR-integrin interactions activate the MAPK cascade, in particular ERK 1/2, with the involvement of non-receptor tyrosine kinase src, tyrosine-kinase family src (Hck, Fgr, Fyn), and focal adhesion-associated protein kinase (FAK) (18). Furthermore, uPAR-mediated cell migration, allowed by uPAR interactions with FPRs and β1 integrins, involves as signaling mediators, specifically, small Rac1 and Rho GTPases (15).

In this study, we investigated FPRs-uPAR-integrin cross-talk as a potential player in ROS generation skin fibroblasts derived from normal subjects and SSc patients.

## Materials and Methods

### Peptides and Chemicals

The hexapeptide Trp-Lys-Tyr-Met-Val-D-Met-NH2 (WKYMVm) was synthesized and HPLC purified (95%) by Innovagen (Lund, Sweden); the peptide uPAR_84–95_ was synthesized by PRIMM (Milan, Italy) and *N*-Formyl-l-methionyl-l-leucyl-l-phenylalanine (fMLF) was obtained from Calbiochem (La Jolla, CA, USA). Protein concentration was determined with a modified Bradford assay (Bio-Rad Laboratories). ECL Plus was obtained from GE Healthcare (Buckinghamshire, UK), and 29, 79-dichlorodihydrofluorescein diacetate (DCHF-DA) was obtained from Molecular Probes (Invitrogen, Paisley, UK). The protease and phosphatase inhibitors cocktail was obtained from Calbiochem. Monoclonal mouse anti-FPR1 phycoerythrin (PE)-conjugated, anti-FPRL1/FPR2 fluorescein (FITC)-conjugated, anti-FPRL2/FPR3 allophycocyanin (APC)-conjugated antibodies were from R&D System (Minneapolis, MN, USA). Mouse anti-gp91^phox^, rabbit anti-p67^phox^, mouse anti-phospho-ERK, and rabbit anti-ERK 2 were from Santa Cruz Biotechnology (Santa Cruz, CA, USA); rabbit anti-actin was obtained from Sigma-Aldrich (St. Louis, MO, USA); mouse anti-uPAR monoclonal antibody R4 was kindly provided by Dr G. Hoyer-Hansen (Finsen Laboratory, Copenhagen, Denmark); secondary anti-mouse and anti-rabbit Abs coupled to HRP were from Bio-Rad (Munchen, Germany). The rabbit antibody anti-uPAR_84–95_ peptide IB (15) was obtained from PRIMM (Milan, Italy), mouse monoclonal anti-uPAR ADG3937 was obtained from American Diagnostica (Greenwich, CT, USA), mouse anti-uPAR monoclonal antibody R3 was kindly provided by Dr G. Hoyer-Hansen (Finsen Laboratory, Copenhagen, Denmark). Diphenyleneiodonium (DPI), PD98059, and NSC23766 were from Calbiochem; P25 peptide and a peptide with the exact composition of amino acids of P25 but in scrambled control peptide (Scp) were from PRIMM; selumetinib (AZD6244) from AstraZeneca; and C37 from the NCI/DTP Open Chemical Repository (Available from: http://dtp.cancer.gov). They were dissolved in dimethyl sulfoxide (DMSO), stored at −20°C, and added to the culture at final concentrations indicated in the text.

### Tissues and Patients’ Samples

Eleven females and three males affected by SSc, admitted to the Department of Translational Medical Sciences of the University of Naples Federico II, were diagnosed by following the ACR/EULAR criteria (23) in limited cutaneous (*n* = 8) or diffuse cutaneous (dcSSc; *n* = 6) subsets (24). The mean age of patients was 49.5 years (range, 30–69 years). Patients according to the disease duration (<5 years for early-stage limited cutaneous SSc and <2 years for early-stage diffuse cutaneous SSc) and skin histopathology were stratified as having an early-stage (*n* = 6) or late-stage (*n* = 8) SSc (25). All patients showing serum positivity for antinuclear Abs (ANA), anti-SCL-70 topoisomerase, and anticentromere (CENP-B) I positivity were included in the study. After obtaining written informed consent, all patients were washed out from steroid treatment at least 30 days before skin biopsy was done. Proton pump inhibitors and vasodilators were allowed. Patients were excluded if severe organ complications prevented steroid treatment washout. All patients with overlapping symptoms of other autoimmune, rheumatic, and/or connective tissue diseases were excluded from the study. Control donors (eight females and two males; mean ± SD age, 45 ± 15 years) were matched with each scleroderma patient (age, sex, and biopsy site) and processed in parallel.

### Cell Cultures

Surgical specimens were mechanically dissociated and trypsinizated, as described previously (26). Cells were plated and cultured in monolayer in DMEM (Life Technologies Carlsbad, CA, USA) supplemented with 10% heat inactivated FBS (Life Technologies), 100 U/ml penicillin G sodium, and 100 mg/ml streptomycin sulfate, at 37°C, in a humidified atmosphere of 5% CO_2_. Fibroblasts from normal subjects and from patients with SSc were used between the 3rd and 10th passage in culture.

The BJ (human foreskin fibroblasts; ATCC accession number CRL-2522), the HGF-1 (human gingival fibroblasts; ATCC accession number CRL-2014), and the MRC-5 (human lung fibroblasts; ATCC accession number CCL-171) were from ATCC (LGC Standards, Milan, Italy) and were grown in DMEM (Life Technologies) with 10% FBS. BJ cells were obtained from ATCC at the sixth passage, subcultured and frozen in stock vials; they were used between the 1st and 10th passage in culture.

The H460 (cell lung cancer; ATCC accession number HTB-177), as a positive control for uPAR expression, was obtained from ATCC and grown in RPMI 1640 medium (Life Technologies) supplemented with 10% FBS.

### Flow Cytometric Analysis of Surface Molecules

Flow cytometric analysis of cell surface molecules was performed as previously described (27). Briefly, after saturation of non-specific binding sites with total rabbit IgG, cells (1 × 10^6^) were incubated for 20 min at +4°C with specific or isotype control antibodies. Finally, cells were washed and analyzed with a FACSCalibur Cytofluorometer using Cell Quest software (Becton & Dickinson, San Fernando, CA, USA). A total of 10^4^ events for each sample were acquired in all cytofluorimetric analyses.

### Western Blot Analysis

Cells were harvested in lysis buffer (50 mM HEPES, 150 mM NaCl, 10% glycerol, 1% Triton X-100, 1 mM EGTA, 1.5 mM MgCl_2_, 10 mM NaF, 10 mM sodium pyrophosphate, and 1 mM Na_3_VO_4_) supplemented with a cocktail of proteases and phosphatases inhibitors. Fifty micrograms of protein was electrophoresed on a 10% SDS-PAGE and transferred onto a polyvinylidene fluoride membrane. The membrane was blocked with 5% nonfat dry milk and probed with specific Abs: mouse anti-uPAR ADG3937 (1 µg/ml), mouse anti-Rac1 (1 µg/ml), mouse anti phospho-ERK (5 µg/ml), rabbit anti-ERK 2 (1 µg/ml), mouse anti-gp91^phox^ (1 µg/ml), rabbit anti-p67^phox^ (1 µg/ml), and rabbit anti-actin (0.5 µg/ml). Finally, washed filters were incubated with HRP-conjugated anti-rabbit or antimouse Abs. The immunoreactive bands were detected by a chemiluminescence kit and quantified by densitometry (ChemiDoc XRS, BioRad) (12).

### ROS Detection

The BJ cells were plated overnight at 2 × 10^4^ cells/well in 96-well plates using DMEM with 10% FBS. Cells were incubated with 5 µM 2′,7′-dichlorodihydrofluorescein diacetate (DCHF-DA) for 30 min in the dark at 37°C. The esterified form of DCHF-DA can permeate cell membranes before being deacetylated by intracellular esterases. The resulting compound, dichlorodihydrofluorescein, reacts with ROS, producing an oxidized fluorescent compound, dichlorofluorescein (DCF), which can be detected by a multiplate reader. After incubation with DCHF-DA, cells were washed twice and treated with medium alone, fMLF (10^−4^ M; 10^−8^ M), uPAR_84–95_ (10^−8^ M), WKYMVm peptide (10^−8^ M), and TGF-β (20 ng/ml) as positive control, in the presence or in the absence of P25 peptide (50 µM), anti-uPAR_84–95_ antibody (5 µg/ml), DPI (10 µM), PD98059 (50 µM), NSC23766 (25 µM), C37 (10 µM), and selumetinib (2.5 µM) for 5, 15, 30, and 60 min at 37°C in a humidified 5% CO_2_ incubator. DCF was detected at a wavelength of 535 nm by a microplate reader (Tecan Trading AG, Switzerland).

### Determination of Rac1 Activity and Association With p67^phox^

BJ cell lysates were *in vitro* treated with GDP and GTPγS (Upstate) to generate Rac1-GDP and Rac1-GTP, respectively. Rac1-GDP and Rac1-GTP containing lysates were precipitated using the p21-binding domain (PBD) of PAK1, bound to agarose beads. Eluted proteins were subjected to SDS-page and Western blot analysis was performed with antip67^phox^, anti-gp91^phox^ antibodies and anti-Rac1 antibody as a control.

BJ cells (1 × 10^6^) were incubated at 37°C with or without specific FPRs agonists for the indicated times. Same amount of total protein from clarified lysates were precipitated using the p21-binding domain (PBD) of PAK1, and eluted proteins were subjected to SDS-page and Western blot analysis was performed using anti-p67^phox^, anti-gp91^phox^ antibodies and anti-Rac1 antibody, as a loading control.

### Histology and Immunohistochemistry

A 3-mm skin punch biopsy was taken from a representative area of 14 SSc patients and from 10 controls. Specimens were fixed in 10% buffered formalin, embedded in paraffin, and serial sectioned (4-mm-thick sections). One section for each case was stained with H&E and the others were stained by immunohistochemistry (streptavidin-biotin standard technique) with the specific primary antibodies (12). Cells showing a definite black staining confined to the nucleus or cytoplasm were judged positive. All slides were examined in a double-blinded fashion by two investigators, and the final staining for each case was expressed as the percentage of positive cells among the total number of counted cells (at least five high-power representative fields).

### Statistical Analyses

All statistical analyses were performed using GraphPad Prism 5.0 software (GraphPad). All the experiments have been executed at least in triplicate. The results are expressed as mean ± SEM. Values from groups were compared using a paired Student’s *t*-test (28). Differences were considered significant when *p* < 0.05.

## Results

### Expression of FPRs and Effects of Their Ligands on ROS Production by Normal Human Fibroblasts

**Figure 1:**
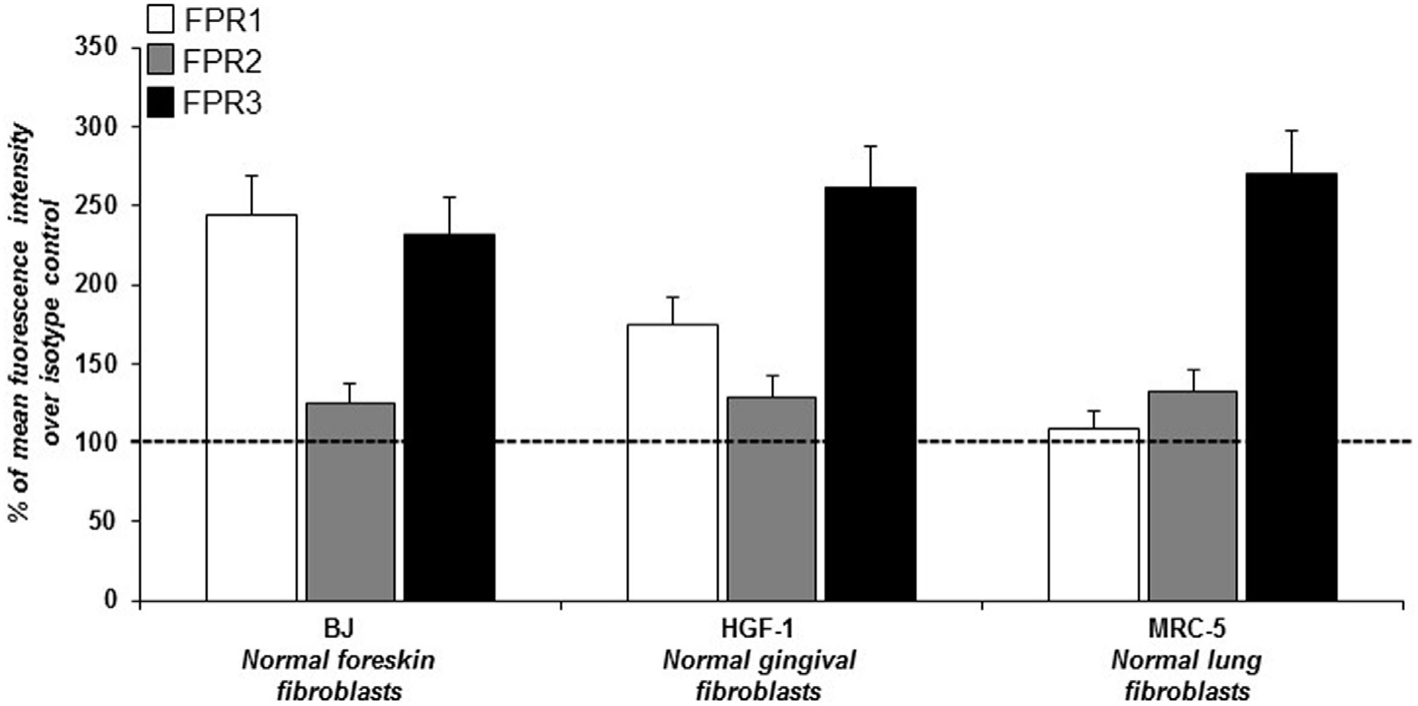
Cytofluorimetric analysis of *N*-formyl peptide receptors (FPRs) expression in human normal fibroblasts. Mean fluorescence intensity of FPR1 (white column), FPR2 (gray column), and FPR3 (black column) expression in BJ (human normal foreskin fibroblasts), HGF-1 (human normal gingival fibroblasts) and MRC-5 (human normal lung fibroblasts) cell lines expressed as a percentage of increase of mean fluorescence intensity of antibody-treated cells over mean fluorescence intensity of isotype control treated cells (considered as 100%).

In order to study ROS generation *via* FPRs-uPAR cross-talk in human fibroblasts, we sought a fibroblast cell line to be used as a model. To this aim we investigated, by cytofluorimetric analysis, FPRs expression in three human fibroblast cell lines from different sources: BJ cells (normal foreskin fibroblasts), HGF-1 cells (normal gingival fibroblasts), and MRC5 cells (normal lung fibroblasts). Fibroblasts from the three cell lines, even with a different pattern of expression, synthesized all the three members of the FPRs family (Figure 1). Given their derivation from human normal foreskin, all the experiments were conducted on BJ non-immortalized fibroblasts capable to proliferate to a maximum of 72 population doublings before the onset of senescence.

We have demonstrated that in normal fibroblasts the FPRs/uPAR cross-talk is able to mediate several functions such as migration, proliferation, and induction of a myofibroblast phenotype through ROS generation, matrix deposition, and α-SMA overexpression (12).

**Figure 2:**
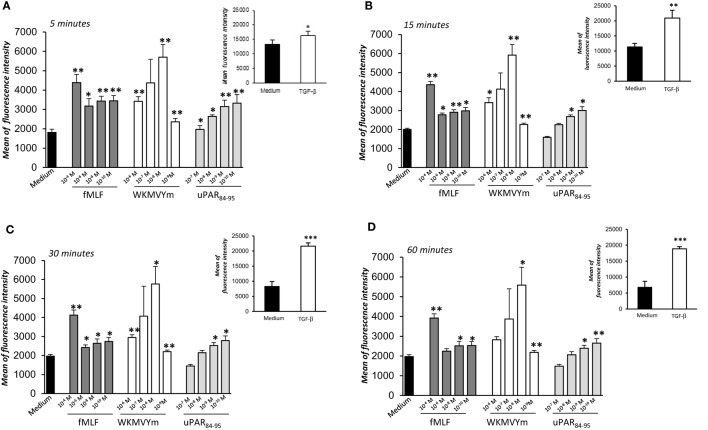
Effects of *N*-Formyl-l-methionyl-l-leucyl-l-phenylalanine (fMLF), WKYMVm peptide, and uPAR_84–95_ on reactive oxygen species (ROS) production in BJ cells. Cells were plated in a 96-well plate and treated with DCHF-DA. At the end of incubation, cells were treated with medium alone (black columns), fMLF (dark gray columns), WKYMVm peptide (white columns), and uPAR_84–95_ peptide (light gray columns). ROS release was measured as dichlorofluorescein (DCF) fluorescence intensity at 5, 15, 30, and 60 min **(A–D)**. Results are expressed as mean fluorescence intensity of DCHF-DA-loaded cells. DCHF-DA-loaded unstimulated cells and TGF-β stimulated cells were examined in parallel, as controls, and are shown in insets. Values are the mean ± SEM of three experiments performed in triplicate. *p < 0.05; **p < 0.001.

We investigated the effects of FPRs activation and cross-talk with uPAR on ROS release from normal fibroblasts, using BJ cells as a model. To this aim, we evaluated ROS levels after stimulation with a wide range of concentrations of specific FPRs agonists, fMLF (10^−4^M–10^−10^M), the synthetic peptide WKYMVm (10^−6^M–10^−9^M), and the synthetic soluble uPAR_84-95_ peptide (10^−7^M–10^−9^M), containing the uPAR-derived ^88^SRSRY^92^ sequence and able to interact with FPRs on the cell surface and to activate their signals. The intracellular ROS levels were determined after 5, 15, 30, and 60 min of stimulation and compared with unstimulated cells (Figures 2A–D).

Figure 2 shows that ROS production from BJ cells was increased in a significant manner after FPRs stimulation with all the three agonists (*p* < 0.05). In particular, we observed that fMLF induced an optimal response both at high concentrations (10^−4^M), which activate the high affinity receptor FPR1, and lower concentrations, active on FPR2. Indeed, the WKMVYm and uPAR_84−95_ peptides exert their effects with a bell-shaped dose response curve, similar to the typical response observed with fMLF in inflammatory cells (27). As a control, the effect of TGF-β (20 ng/ml), able to stimulate ROS release by a FPRs independent pathway (4), was examined in parallel in BJ cells (Figure 2, panel A–D; insets).

### Role of FPRs-uPAR-Integrin Cross-Talk in ROS Generation by Normal Human Fibroblasts

**Figure 3:**
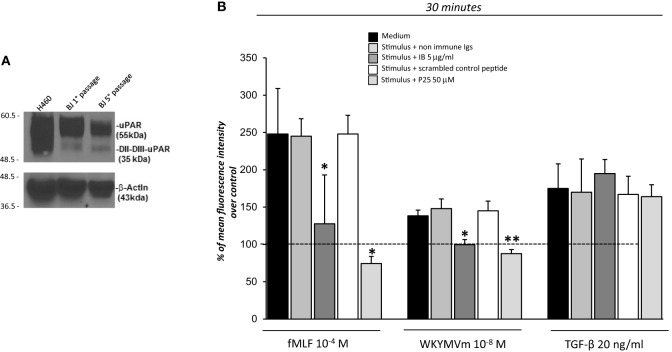
Effects of the inhibition of the cross-talk between FPRs-uPAR-Integrins on reactive oxygen species (ROS) induction in BJ cells. **(A)** Western blot analysis of uPAR expression in H460 cell line as a positive control (lane 1), BJ cells at first passage (lane 2) and fifth passage (lane 3) in culture with the R4 anti-uPAR mAb and with an anti-β-actin Ab, as a loading control. **(B)** BJ cells were plated in a 96-well plate and treated with DCHF-DA. At the end of incubation, cells were treated with medium alone, *N*-formyl-l-methionyl-l-leucyl-l-phenylalanine (fMLF), WKYMVm or TGF-β in the absence (black columns) or in the presence of nonimmune immunoglobulins (medium gray columns), IB antibody (dark gray columns), scrambled control peptide (Scp) (white columns), and P25 peptide (light gray columns). ROS release was measured as dichlorofluorescein (DCF) fluorescence at 30 min. Results are expressed as a percentage of increase of mean fluorescence intensity of stimulated DCHF-DA-loaded cells in respect to unstimulated DCHF-DA-loaded cells (considered as 100%). Values are the mean ± SEM of three experiments performed in triplicate. *p < 0.05; **p < 0.001.

The observation that ^88^SRSRY^92^ stimulates ROS production (Figure 2) suggests that FPRs cross-talk with cell surface uPAR may mediate the same effect. Indeed, uPAR is an important signaling partner of FPRs at the cell-surface (21). Moreover, several studies show that uPAR also requires integrins as co-receptors (15). In fact, uPAR over-expression in tumor cells, controls cell migration and invasion by the recruitment of integrins and FPR1 on cell surface and regulating their signaling pathways (15). Thus, we first investigated the expression of uPAR, both in the native and in the cleaved form (DII-DIII-uPAR), by Western blot, in BJ cells at different time of culture (first and fifth passage). Figure 3A shows that BJ cells markedly over-expressed uPAR in the native form; DII-DIII-uPAR was also expressed although to a lesser extent (lane 2 and 3).

Then to test the hypothesis that FPRs could regulate ROS production in fibroblasts through the recruitment, at cell surface of the uPAR/integrins complex, we performed the ROS production assay in the presence of IB, a polyclonal antibody directed against the region involved in uPAR interaction with FPRs, corresponding precisely to the ^88^SRSRY^92^ region (17) and in the presence of the P25 peptide, which disrupts uPAR interactions with β1 or β2 integrins (29).

Treatment of BJ cells with the IB antibody (5 µg/ml) and the P25 peptide (50 µM) completely inhibited FPRs-mediated ROS production, in response to their specific agonists, fMLF 10^−4^M and WKYWM peptide 10^−8^M, whereas non-immune immunoglobulins and a Scp did not exert any effect. As a control, the IB antibody and the P25 peptide did not affect TGF-β induced ROS release by BJ cells (Figure 3B).

These results suggest that FPRs could control ROS production by interacting with uPAR, thus participating to a supramolecular complex including integrins, as already demonstrated for cell migration and invasion (15).

### Rac1 and ERK1/2 Role in FPRs-Mediated ROS Production in Normal Human Fibroblasts

In order to study the signaling pathways involved in FPRs-mediated ROS generation in fibroblasts, we focused on the small GTPase Rac1 and on the ERK1/2 pathway.

uPAR-mediated cell migration, allowed by uPAR interactions with FPRs and β1 integrins, involves as signaling mediators specifically small Rac1 and Rho GTPases (15).

One important effector of Rac1 activity is p67^phox^, which combines with other components of the NADPH oxidase system to generate a functional complex for producing ROS (30). NADPH oxidase system-derived ROS can also activate other downstream signals, such as ERK 1/2 signaling pathways (9). Interestingly, also uPAR-dependent signaling pathways lead to the activation of ERK MAPKs through the activation of PI3K (18).

**Figure 4:**
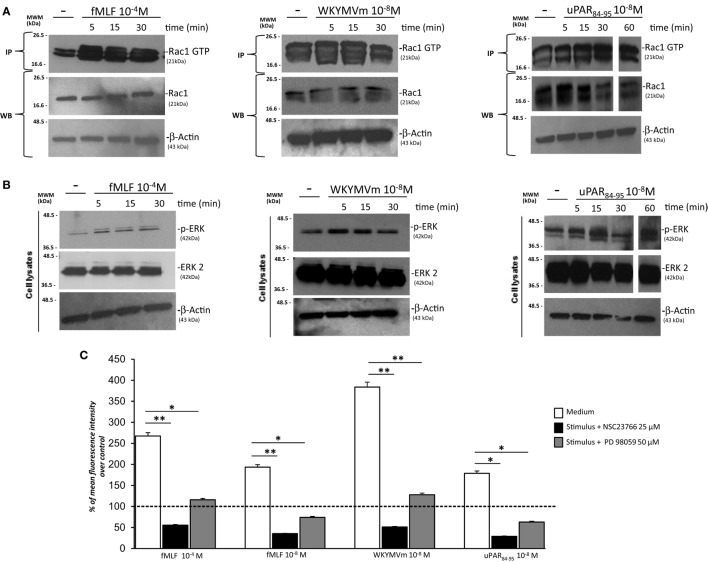
FPRs-mediated Rac1 and ERK activation in BJ cells. **(A)** BJ cells, treated with medium alone (-),*N*-formyl-l-methionyl-l-leucyl-l-phenylalanine (fMLF), WKYMVm peptide, or uPAR_84–95_ peptide, were lysed and subjected to Rac1 activity assay using PAK-PBD-glutathione sepharose beads. Immunoprecipitates and the corresponding total lysates, as an input control, were subjected to Western blot analysis with an anti-Rac1-specific Ab and with an anti-β-actin Ab, as a loading control. **(B)** BJ cells, treated with medium alone, fMLF, WKYMV peptide, or uPAR_84–95_ peptide were lysed and subjected to Western blot analysis with an anti phospho-ERK 1/2 (p-ERK)-specific Ab and then with anti ERK-2 and anti-β-actin Abs, as a loading control. **(C)** BJ cells were plated in a 96-well plate and treated with DCHF-DA. At the end of incubation, cells were treated with fMLF, WKYMVm peptide, and uPAR_84–95_ peptide in the absence (white columns) or in the presence of NSC23766 (black columns) or PD98059 (gray columns). ROS release was measured as dichlorofluorescein (DCF) fluorescence intensity at 5 min. Results are expressed as a percentage of increase of mean fluorescence intensity of stimulated DCHF-DA-loaded cells in respect to unstimulated DCHF-DA-loaded cells (considered as 100%). Values are the mean ± SEM of three experiments performed in triplicate. *p < 0.05; **p < 0.001.

We analyzed the effects of FPRs stimulation with optimal concentrations of fMLF (10^−4^M), WKYMVm peptide (10^−8^M), and uPAR_84–95_ (10^−8^M) on Rac1 and ERK1/2 activation. All the stimuli increased the levels of Rac1-GTP and p-ERK 1/2 (Figures 4A,B). Furthermore, we evaluated ROS levels after stimulation with the same agonists, in the absence or in the presence of an inhibitor of Rac-specific GEF (guanine nucleotide exchange factor) Trio and Tiam1 (NSC23766) (25 µM) and of a specific MEK 1/2 inhibitor (PD98059) (50 µM). BJ cells, which responded to all the three stimuli, were unable to produce ROS in presence of NSC23766 and PD98059 (Figure 4C). These results are compatible with the hypothesis that FPRs stimulation in normal fibroblasts determines ROS production by activating Rac1- and ERK 1/2-dependent signals.

### FPRs-Mediated ROS Production and NADPH Oxidase-2 Activation in Normal Human Fibroblasts

**Figure 5:**
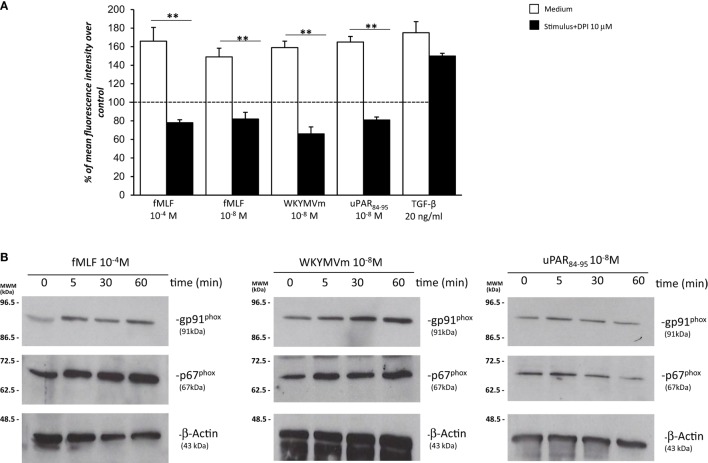
Effects of the NOX-inhibitor diphenyleneiodonium (DPI) on *N*-formyl peptide receptor (FPR)-mediated reactive oxygen species (ROS) production and FPRs-induced modulation of gp91^phox^ and p67^phox^ expression in BJ cells. **(A)** BJ cells were plated in a 96-well plate and pretreated for 1 h at 37°C with 10 µM of the NOX-inhibitor DPI. At the end of incubation, cells were treated with *N*-formyl-l-methionyl-l-leucyl-l-phenylalanine (fMLF), WKYMVm peptide, uPAR_84–95_ peptide, or TGF-β. Results are expressed as percentage of increase of mean fluorescence intensity of stimulated DCHF-DA-loaded cells in respect to unstimulated DCHF-DA-loaded cells (considered as 100%). Values are the mean ± SEM of three experiments performed in triplicate. **p < 0.001. **(B)** Western blot analysis with anti**-**gp91^phox^, **-**p67^phox^, and β-actin antibodies of lysates from BJ cells stimulated with fMLF, WKYMVm peptide, and uPAR_84–95_ peptide for 0, 5, 30, and 60 min.

Having established that FPRs activation induced ROS production through Rac1 and ERK 1/2 signaling in BJ cells, we assessed whether ROS were generated by the NADPH oxidase complex through cell pretreatment with the NOX-inhibitor DPI (10 µM, 1 h) before the addition of fMLF (10^−4^M), WKYMVm peptide (10^−8^M), and uPAR_84–95_ (10^−8^M). Figure 5A shows that treatment with DPI in BJ cells determined a significant reduction in ROS levels compared to untreated cells (*p* < 0.001).

Diphenyleneiodonium treatment did not significantly affect ROS release by BJ cells (Figure 5A). Indeed, in fibroblast cells, TGF-β mostly induces mitochondrial ROS through the complex III of the electron transport chain (31).

Recently, it has been demonstrated that in SSc fibroblasts, NOX2 and NOX4 are constitutively overexpressed and are responsible for ROS production in these cells (9).

To investigate whether FPRs stimulation could induce ROS production through upregulation and/or activation of the NOX2 complex, we evaluated, by Western blot, the expression levels of gp91^phox^ and p67^phox^ after stimulation with specific agonists of FPRs. Figure 5 shows that BJ cells responded to fMLF (10^−4^M), WKYMVm peptide (10^−8^M), and uPAR_84–95_ (10^−8^M) by slightly upregulating the expression of both gp91^phox^ and p67^phox^ (Figure 5B).

**Figure 6:**
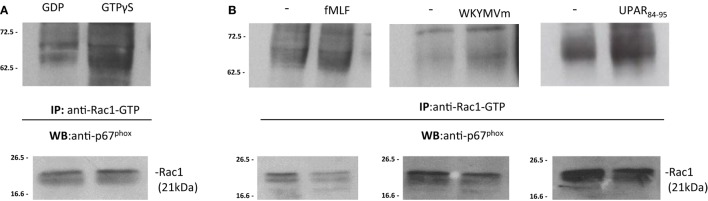
FPRs-mediated Rac1 GTP-p67^phox^ interaction in BJ cells. **(A)** BJ cells lysates were treated with GDP and GTPγS (Upstate) to generate Rac1-GDP and Rac1-GTP, respectively. Rac1-GDP and Rac1-GTP containing lysates were precipitated using the p21-binding domain (PBD) of PAK1, bound to agarose beads. Eluted samples and the corresponding total lysates were subjected to Western blot analysis with a polyclonal anti-p67^phox^ antibody and with anti-Rac1 antibody as a loading control, respectively. **(B)** BJ cells, after incubation with medium alone (-),*N*-formyl-l-methionyl-l-leucyl-l-phenylalanine (fMLF), WKYMVm peptide, for 5 min, or uPAR_84–95_ (10^−8^M) for 30 min at 37°C in a humidified (5% CO_2_) incubator, were lysed and subjected to Rac1 activity assay. Active Rac1 (Rac1-GTP) was precipitated from cell lysates using the PBD of PAK1, bound to agarose beads. Eluted samples and the corresponding total lysates were subjected to Western blot analysis with a polyclonal anti-p67^phox^ antibody and with anti-Rac1 antibody as a loading control, respectively.

Upon stimulation, activated GTP bound-Rac1 and/or Rac2 translocate to the plasma membrane and recruit p67^phox^ by binding to its N-terminal (30, 32). In order to demonstrate that the binding of p67^phox^ to Rac1/2-GTP is the limiting step in the assembly of the active NADPH oxidase complex, BJ cell lysates were treated with GDP and GTPγS to generate Rac1-GDP and Rac1-GTP, respectively. Cell lysates containing Rac1-GDP and Rac1-GTP were incubated with the p21-binding domain (PBD) of PAK1, bound to agarose beads. Western blot analysis of precipitated with a polyclonal anti-p67^phox^ antibody revealed that Rac1-GTP binds to p67^phox^ (Figure 6A). As a control, Western blot analysis with anti-gp91^phox^ antibody did not show any association with Rac1-GTP (not showed).

To investigate whether FPRs stimulation could induce the interaction between Rac1-GTP and p67^phox^, the same pull-down experiments were carried out in BJ cells, after stimulation with the specific FPRs agonists, fMLF (10^−4^M), WKYMVm peptide (10^−8^M), and uPAR_84–95_ (10^−8^M). Western blot analysis of precipitated polyclonal anti-p67^phox^ antibody revealed that FPRs stimulation increased interaction between Rac1-GTP and p67^phox^, thus allowing the assembly of the active NOX2 complex (Figure 6B) (33).

After stimulation with specific FPRs agonists, association between Rac1-GTP and gp91^phox^ did not increase (not showed). Thus, our data demonstrate that FPRs/uPAR-mediated ROS generation in fibroblast cells is mediated by a direct binding of Rac1 to p67^phox^ that, in turn, could interact with gp91^phox^ and probably p22^phox^ membrane subunits to generate the active NOX2 complex as described (33).

### *Ex Vivo* Expression of Different uPAR Forms in Human Fibroblasts

Urokinase receptor cleavage contributes to the impaired angiogenesis observed in SSc patients (34, 35). uPAR gene inactivation causes dermal and pulmonary fibrosis and peripheral microvasculopathy in mice. Moreover, native full-length uPAR expression is significantly decreased in the skin of SSc patients, as assessed by a monoclonal anti-uPAR/domain DI antibody (36).

**Figure 7:**
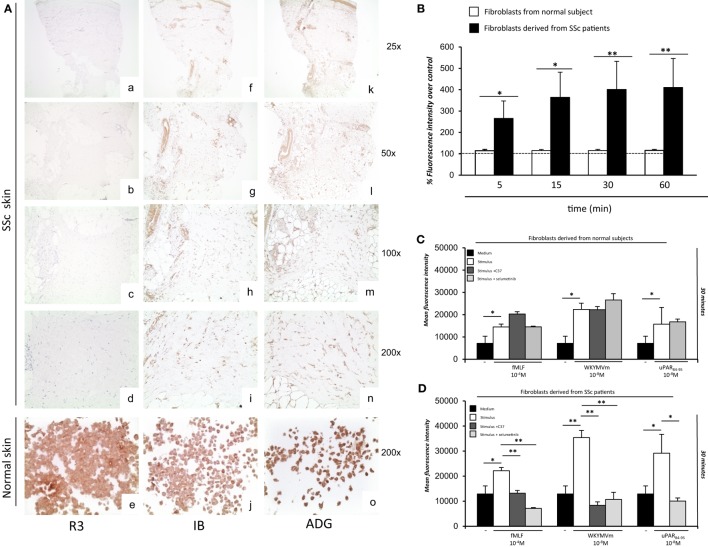
*Ex vivo* expression of uPAR forms and analysis of the effects of C37 and selumetinib on FPRs-mediated reactive oxygen species (ROS) production in fibroblasts derived from normal subjects and systemic sclerosis (SSc) patients. **(A)** (a–d) staining for R3 in localized scleroderma: fibroblasts, lymphocytes, and endothelial cells were negative for R3 (a: original magnification, ×25; b: original magnification, ×50, c: original magnification, ×100; d: original magnification, ×200); (e) staining for R3 in normal human fibroblast cell line: fibroblasts were positive for this antibody [original magnification, ×200] (f–i) staining for IB in localized scleroderma: most fibroblasts, lymphocytes and endothelial cells were positive for IB (f: original magnification, ×25; g: original magnification, ×50, h: original magnification, ×100; i: original magnification, ×200); (j) staining for IB in normal human fibroblast cell line: fibroblasts were positive for this antibody (original magnification, ×200) (k–n) staining for ADG3937 in localized scleroderma: positivity for ADG3937 was observed in fibroblasts, lymphocytes and endothelial cells (k: original magnification, ×25; l: original magnification, ×50, m: original magnification, ×100; *n* original magnification, ×200); (o) staining for ADG3937 in normal human fibroblast cell line: fibroblasts were positive for this antibody (original magnification, ×200) **(B)** Fibroblasts derived from normal subjects (white columns) and SSc patients (gray columns) were plated in a 96-well plate and treated with DCHF-DA. At the end of incubation, cells were washed, and ROS release was measured as dichlorofluorescein (DCF) fluorescence intensity. Results are expressed as a percentage of increase of mean fluorescence intensity of DCHF-DA–loaded cells, as compared with DCHF-DA–unloaded cells (considered as 100%). Values are the mean ± SEM of three experiments performed in triplicate. *p < 0.05; **p < 0.001. **(C)** Fibroblasts derived from primary cultures of three normal subjects were plated in a 96-well plate and treated with DCHF-DA. At the end of incubation, cells were treated with fMLF, WKYMVm peptide, or uPAR_84–95_ peptide in the absence (white columns) and in the presence of C37 (medium gray columns) or selumetinib (light gray columns). ROS release was measured as dichlorofluorescein (DCF) fluorescence intensity at 30 min. Values are the mean ± SEM of three experiments performed in triplicate. *p < 0.05. **(D)** Fibroblasts derived from primary cultures of three SSc patients were plated in a 96-well plate and treated with DCHF-DA. At the end of incubation, cells were treated with *N*-formyl-l-methionyl-l-leucyl-l-phenylalanine (fMLF), WKYMVm peptide, or uPAR_84–95_ peptide in the absence (white columns) and in the presence of C37 (medium gray columns), selumetinib (light gray columns). ROS release was measured as DCF fluorescence intensity. Values are the mean ± SEM of three experiments performed in triplicate. *p < 0.05; **p < 0.001.

Although full-length uPAR expression is downregulated in SSc dermis, we hypothesized that the DII-DIII-uPAR_88–92_ form, able to interact with FPRs, could instead be increased. To confirm our hypothesis, we investigated the expression of the different uPAR forms in SSc and normal skin biopsies by IHC. In particular, we used the R3 mAb that recognizes domain DI (37), thus the full-length uPAR; the IB polyclonal antibody specifically directed against the ^88^Ser-Arg-Ser-Arg-Tyr^92^ sequence of uPAR, which is mostly exposed only in the truncated uPAR form (38); the ADG3937 mAb, recognizing an epitope located in the domains DII + DIII which, also, identifies the full-length receptor. SSc skin biopsies revealed dermal fibrosis showing prominent involvement of the deep dermis and the subcutaneous fat, admixed with chronic inflammatory infiltrate, mostly confined deeply around vessels of the subcutis. All biopsies showed an absent/low staining for R3 in fibroblasts, endothelial cells, and lymphocytes. Conversely, IB was found positive in fibroblasts, endothelial cells, and lymphocytes of all specimens; finally, ADG3937 was expressed only in 4 out 14 selected cases. By contrast, skin fibroblasts from control donors showed positivity for the three antibodies (Figure 7A).

### Effect of Selumetinib and C37 on FPRs/uPAR-Mediated ROS Production in Fibroblasts Derived From SSc Patients

We next aimed to investigate whether inhibition of the structural and functional interaction between FPRs and uPAR by new compounds or tested drugs could affect ROS generation in fibroblasts from SSc patients.

First, fibroblasts derived from primary cultures of three healthy controls and three SSc patients were tested for their ability to produce ROS in basal conditions (Figure 7B). To this aim, fluorescence was measured in both cell types after loading with DCHF-DA without any stimulation. Fibroblasts from SSc patients showed an increased level of basal ROS generation at all time points examined, as compared to normal primary fibroblasts.

Then, fibroblasts derived from primary cultures of three healthy controls (Figure 7C) and three SSc patients (Figure 7D) were tested for their ability to produce ROS after stimulation with fMLF (10^−4^M), WKYMVm (10^−8^M) peptide and uPAR_84–95_ (10^−8^M) peptide, in the absence or in the presence of C37 (10 µM) and selumetinib (2.5 µM). C37 is a small molecule identified by our group by structure-based virtual screening able to target the ^88^SRSRY^92^ sequence of uPAR in the hot-spot residue Arg^91^ (39); for this reason, its effect on FPRs/uPAR-mediated ROS production was evaluated after stimulations with fMLF and WKYMVm. The effect of selumetinib, a highly selective MEK1 inhibitor currently approved for various anticancer therapies (40), on ROS release mediated by FPRs/uPAR activation was evaluated in parallel, after all the three stimuli. Indeed, we have demonstrated ERK 1/2 involvement in ROS generation induced by FPRs/uPAR activation.

Figure 7C shows that in primary fibroblasts from healthy controls, which significantly responded to all the three stimuli, neither C37 nor selumetinib were able to inhibit FPRs/uPAR-mediated ROS production.

Figure 7D shows that in primary fibroblasts from SSc patients, which responded more efficiently than normal fibroblasts to all the three stimuli, both C37 and selumetinib were significantly active.

The results from these studies show that C37 and selumetinib, through inhibition of FPRs-mediated ROS generation, may lead to the development of novel antifibrotic therapeutic strategies.

## Discussion

*N*-formyl peptide receptors involvement in different inflammatory conditions and in innate immune responses is well established (11). We have already demonstrated that FPRs and their cross-talk with uPAR are involved in the pathogenesis of SSc. Here, we investigated whether FPRs stimulation and their cross-talk with uPAR could induce ROS generation in fibroblasts, thus playing a role in some ROS-mediated processes such as tissue remodeling and fibrosis.

In order to study the molecular mechanisms of FPRs-mediated ROS generation in fibroblast cells, we pursued our studies on the BJ cell line. We demonstrated that FPRs stimulation induces ROS generation in fibroblasts by interacting with uPAR and integrins, as shown in epithelial cell migration and invasion (15). Our study also reported that FPRs/uPAR/β1integrin cross-talk determines ROS production through Rac1 and ERKs activation in human skin fibroblasts.

One important effector of Rac1 activity is p67^phox^, which combines with the NADPH oxidase system to generate a functional complex for producing ROS. Upon activation by GTP-Rac1, p67^phox^ translocates to the membrane where it associates with gp91^phox^. FPRs stimulation promoted gp91^phox^ and p67^phox^ expression as well as a direct interaction between GTP-Rac1 and p67^phox^.

We already provided *in vitro* and *in vivo* evidence that human normal skin fibroblasts expressed FPRs and that SSc fibroblasts overexpress these receptors (12). Here, we evaluated the role of FPRs interaction with uPAR and integrins in the pathogenesis of SSc.

It has been reported that skin sections from uPAR-deficient mice showed increased dermal thickness, collagen content and a significantly greater myofibroblast count than uPAR wild-type mice, mimicking the histopathological features of SSc. Moreover, the expression of full length uPAR was decreased in skin biopsies of SSc patients (36). Since the cleavage of uPAR is crucial in fibroblast-to-myofibroblast transition (12, 41) and has been implicated in SSc microvasculopathy (34), we hypothesized that the DII-DIII-uPAR_88–92_ form, able to interact with FPRs, could instead be increased. To confirm our hypothesis, we analyzed the expression of the different uPAR forms on SSc skin biopsies. We demonstrated that SSc fibroblasts showed increased membrane levels of DII-DIII-uPAR_88–92_, expressing at the N-terminus the chemotactic sequence able to interact with overexpressed FPRs.

According to their increased expression levels, *in vitro* treatment of SSc fibroblasts with C37, a new small molecule able to inhibit the cross-talk between FPRs and uPAR, inhibited ROS production, after stimulation of FPRs with specific ligands. Inhibition of the MAPK/ERK pathway with selumetinib also blocked ROS production upon FPRs stimulation in the same cells.

In conclusion, the results of the present study show that FPRs, through the interaction with the uPA/uPAR system and integrins, induce increased levels of ROS. Both FPRs and a cleaved form of uPAR were able to interact with FPRs, which appear to be overexpressed in skin biopsies of SSc patients when compared to normal controls. This observation reinforces our previous hypothesis of the possible involvement of FPRs/uPAR in the pathogenesis of SSc. Since FPRs functional interaction with uPAR and their signal can be efficiently inhibited by new small molecules, our observations can also help in the development of novel therapeutic strategies in the treatment of SSc.

## Ethics Statement

The study was conducted on samples already obtained for diagnostic purposes, and all patients signed a written informed consent.

## Author Contributions

FN: responsible for cell cultures, responsible for cellular treatments, WB, Co-IP, and was a contributor in writing the manuscript; FWR: performed cytometric analysis and ROS assay, analyzed the data, and was a contributor in writing the manuscript AP: performed cytometric analysis and ROS assay; SV, GI, MM, SS: performed immunohistochemistry analysis; AL, PR, CS, GM, MMC, AdP: performed data analysis; NM: analyzed the data and was the main contributor in writing the manuscript.

## Conflict of Interest Statement

The authors declare that the research was conducted in the absence of any commercial or financial relationships that could be construed as a potential conflict of interest.
